# Dynamic Multi-Behaviour, Orientation-Invariant Re-Identification of Holstein-Friesian Cattle

**DOI:** 10.3390/s25102971

**Published:** 2025-05-08

**Authors:** Maarten Perneel, Ines Adriaens, Jan Verwaeren, Ben Aernouts

**Affiliations:** 1 Department of Biosystems, Division of Animal and Human Health Engineering, KU Leuven, Campus Geel, Kleinhoefstraat 4, 2440 Geel, Belgium; 2 Department of Data Analysis and Mathematical Modelling, Faculty of Bioscience Engineering, Ghent University, Coupure Links 653, 9000 Ghent, Belgium

**Keywords:** re-identification, pose estimation, behaviour classification, cattle

## Abstract

To perform reliable animal re-identification, most available algorithms require standardised animal poses. However, this lack of versatility prevents widespread application of these algorithms in behavioural research and commercial environments. To circumvent this, we incorporated information about the orientation and behaviour of the animals in an embedding-based algorithm to re-identify Holstein-Friesian cattle. After all, the orientation and behaviour of an animal determine which body parts of an animal are visible from the camera’s perspective. We evaluated our approach using a dataset with more than 11,000 instance segments of Holstein-Friesian cattle, but our methodology is readily generalisable to different animal species. Our results show that incorporation of informative metadata parameters in the re-identification procedure increases the rank-1 re-identification accuracy from 0.822 to 0.894, corresponding to a 40% reduction in the number of incorrectly identified animals.

## 1. Introduction

In modern cattle farming, animal identifiability is essential for registering animal performance, health, and welfare in the context of good animal management. To this end, all animals on a farm are identified regularly, whether it is to record milk production data, provide individualised amounts of concentrate, keep track of the growth of youngstock, etc. Various affordable and effective methodologies to identify cattle by human handlers were developed in the past, such as ear tags, collars, tattoos, brand marks or RFID tags [1,2]. However, when studying cattle behaviour using video monitoring, none of these methods provides sufficient robustness to assure continuous identifiability of the animals. This is because it is relatively easy for the animals to strike a pose in which none of the multiple possible identification marks are visible. Moreover, all of the previously specified identification methods require to modify the animal in some way. Hence, none of these methods is used by the animals themselves to recognise herdmates. Nevertheless, the presence of a linear social hierarchy in cattle [3] implies the identifiability of the individuals constituting a social group. Since, in contrast to pigs [4,5], cows only rarely use sound to communicate, they rely mainly on visual and olfactory cues to recognise each other [6]. Among those two ’natural’ methods, vision has the most potential to develop a robust and automated re-identification system for cattle that could work from any perspective as soon as there is sufficient light to distinguish the animals from the surrounding environment.

Compared to applications and developments in humans, animal re-identification is still in its infancy. In human re-identification, large datasets are available, such as Market-1501 [7], DukeMTMC [8], and MSMT17 [9]. This availability of large datasets allows the development of complex, high-performing re-identification algorithms. For example, for the Market-1501 dataset [7], which contains 32,000 images of 1501 different identities, collected using six different cameras, rank-1 re-identification accuracies over 0.95 are reported by Chen et al. [10], Wang et al. [11], and Zhang et al. [12]. To improve generalisability and to reduce the number of incorrect re-identifications, current research focuses on the re-identification of occluded persons [12], usage of attention mechanisms [10], and non-supervised (pre-)training [13]. In contrast to human applications, data availability is often a limiting factor in developing highly versatile and practical algorithms for animal re-identification. In animal re-identification, most datasets only contain a limited number of animals and a limited number of images per animal. For example, the OpenCows2020 dataset of Andrew et al. [14] includes 4736 images of 46 cows. Additionally, most studies use a standardised pose. For example, in the case of standing animals, being either top views [14], front views [15], lateral views or a combination of two of the aforementioned poses [16], but never a variable animal pose.

Using a variable pose, however, adds significant complexity to the re-identification task. For example, if an animal is viewed from the side, oriented with its head to the right, one primarily observes the right flank of the animal. If the animal turns around 180°, one will primarily observe the left flank of the animal. Due to the randomness in the pied patch pattern, especially in Holstein-Friesian cattle, the left and right flank of the animal may show quite a different patch pattern. Therefore, re-identifying an animal showing its left flank by using reference images of the right flank of animals will give poor results. Previous research handled this issue by standardising the pose [17] or using a perfect top view of the animals [14,18,19]. This approach is, however, detrimental for the application of computer vision-based re-identification of cattle in commercial environments, where the ability to cope with a large variability in animal poses is an essential condition for the broad applicability of computer vision-based re-identification. As state-of-the-art methods do not meet this prerequisite, the potential use of computer vision-based re-identification on dairy farms is currently limited to the milking parlour/robot [18], separation gates [20], and concentrate stations. However, at these locations, the added value of computer vision-based re-identification is limited compared to highly cost-effective RFID-based re-identification.

To overcome the hurdles mentioned above, we aimed to develop an embedding-based re-identification algorithm [16,21] able to handle all possible poses which could be shown by cattle. Therefore, we combined two neural networks: a neural network performing simultaneous pose-estimation and behaviour classification, and a neural network which generates embeddings in a versatile way. We use an embedding-based approach rather than a classification-based method since the former obviates the need to retrain and redesign the neural network every time a new animal enters the herd. By combining the results of both networks, we can re-identify an animal, conditional on its behaviour and orientation with respect to the camera, obviating the need for a standardised animal pose.

## 2. Materials and Methods

### 2.1. Data

For this study, we collected data at a commercial Belgian dairy farm. Between December 2021 and October 2023, we monitored a pen housing eight heifers aged 8 to 12 months. During the experimental period, six groups of animals were monitored, resulting in a total of 48 animals. Video footage was collected continuously during day and night with a roof-mounted 4MP camera (Hikvision Europe, Hoofddorp, The Netherlands, model DS-2CD2643G2-IZS) at 2 fps. For this study, only daylight video recordings were retained.

Of the 48 monitored animals, 29 were purebred red Holstein-Friesian and 19 were purebred Holstein-Friesian. Animals were subdivided into six coat pattern categories based on their main colour (red or black) and the amount of white in the coat. For black Holstein animals, animals with less than 20% white in their coat are considered black (b), animals which have between 20% and 80% white in their coat are considered black and white (bw), and animals which have more than 80% white in their coat are considered white and black (wb). Analogously, red Holstein animals are categorised into red (r), red and white (rw), and white and red (wr) animals. Table 1 shows the frequency of each of these six coat patterns in the dataset.

### 2.2. Instance Segmentation

To obtain instance embeddings (Section 2.5) containing only information about a single animal, instance segmentation of video frames is necessary. For instance segmentation, we used a Mask-RCNN neural network [22], which was finetuned for optimal segmentation of cattle using the CoBRA_segmentation_youngstock dataset (https://doi.org/10.5281/zenodo.14975828).

### 2.3. Pose-Estimation and Behaviour Classification

**Figure 1:**
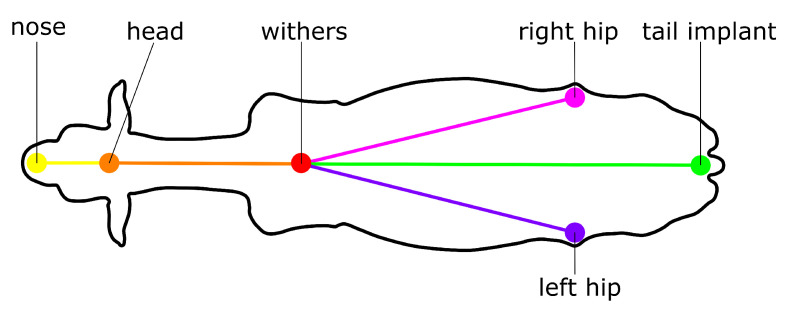
Overview of the skeleton used for pose-estimation.

Our re-identification methodology (Section 2.6) combines instance embeddings with metadata providing more information about which body parts of an animal are visible and from which perspective. In this paper, we use the animal’s orientation and behaviour as informative metadata parameters and determine them using pose-estimation and behaviour classification. We used the animal skeleton presented in Figure 1 for pose-estimation. In this skeleton, the withers are the central keypoint, to which all other keypoints are connected in a hierarchical manner. With regard to the animal’s behaviour, we distinguished three possible behaviours: lying on the left side (lying left), lying on the right side (lying right), and standing.

**Figure 2:**
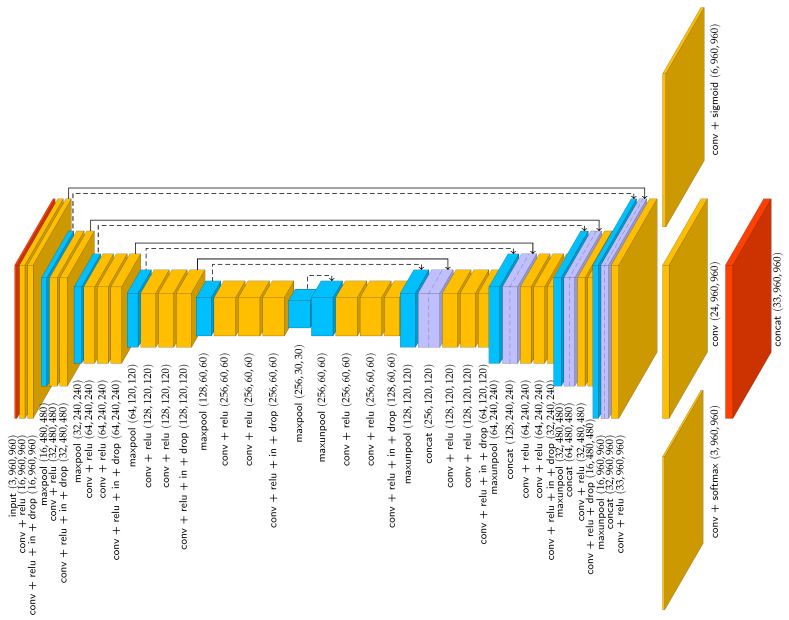
Visualisation of the used neural network architecture for simultaneous pose-estimation and behaviour classification. Specified tensor sizes are indicative for a 960×960 image. Solid arrows represent tensor transfer, dashed arrows represent transfer of maxpool indices. conv = separable convolution, in = instance normalisation, drop = dropout, concat = concatenation.

To enable simultaneous pose-estimation and behaviour classification, we used the model of Perneel et al. [23] but extended the output with three channels (Figure 2). Each of these three extra channels represents a specific behaviour: lying left (ll), lying right (lr), and standing (s). By applying a softmax activation function on these behavioural channels, their joint output corresponds to a probability distribution. During training, when constructing the ground truth network output *Y*, a pixel of the channel corresponding with behaviour b∈B={ll,lr,s} takes on a value of one if the keypoint heatmap of the central keypoint (the withers) has a probability above 0.2 and the corresponding animal displays behaviour *b*. Under all other conditions, pixel values are set to zero. As such, during inference, the behavioural channels predict the animal’s behaviour conditionally on the presence of a central keypoint.

The original loss function of Perneel et al. [23] consisted of two terms: the “location loss” and the “association loss”. The former quantifies the loss of the output channels responsible for detecting individual keypoints. The latter quantifies the loss of the output channels used to associate keypoints of different classes into skeletons representing animals. To also train the behavioural channels, the original loss function was extended with a term called “behaviour loss”, resulting in the loss function Equation (1). The behaviour loss is inspired by the cross-entropy loss commonly used for training classification neural networks [24,25,26].

(1) $$\begin{array}{cc} L= & \;\beta_{1}\underset{\mathrm{Location}\;\mathrm{loss}}{\underbrace{\frac{1}{wh|P|}\sum_{i\in P}\left|\right|{\hat{Y}}_{i}-Y_{i}{\left|\right|}^{2}}}\\ \; & +\beta_{2}\underset{\mathrm{Association}\;\mathrm{loss}}{\underbrace{\frac{1}{1+\sum_{i\in A}∥1\left[Y_{i}\neq 0\right]∥}\sum_{i\in A}{∥1\left[Y_{i}\neq 0\right]⊙\left(\frac{{\hat{Y}}_{i}-Y_{i}}{\gamma }\right)∥}^{2}}}\\ \; & -\beta_{3}\underset{\mathrm{Behaviour}\;\mathrm{loss}}{\underbrace{\frac{1}{1+\sum_{i\in B}∥1\left[Y_{i}\neq 0\right]∥}\sum_{i\in B}\sum_{j=1}^{h}\sum_{k=1}^{w}Y_{ijk}\log\left({\hat{Y}}_{ijk}+(1e-10)\right)}} \end{array}$$

In Equation (1), *w* and *h* are the width and height of the image, and ⊙ is used to represent an element-wise matrix multiplication. Furthermore, *P* is used to denote the indices of the (6) channels that represent the keypoint probability maps, *A* to denote the indices of the (24) keypoint association maps, and *B* to denote the (3) channels used for behaviour prediction. The network for pose-estimation and behaviour classification was trained on the CoBRA_pose_behaviour_youngstock dataset (https://doi.org/10.5281/zenodo.15016853) for 100 epochs of 1000 batches of a single image. During the initial 50 epochs, $\beta_{1}$, $\beta_{2}$, and $\beta_{3}$ were set to one. During the last 50 epochs, $\beta_{1}$, $\beta_{2}$, and $\beta_{3}$ were adapted every tenth epoch so that each of the three terms in Equation (1) has an average value of 1/3. By doing so, each loss term contributes equally to the total loss and $\bar{L}=1$.

### 2.4. Data Processing

To generate instance segments and accompanying metadata (animal orientation and behaviour), frames were sampled randomly from the collected video recordings. After applying a static mask to occlude neighbouring pens, frames were processed employing instance segmentation, pose-estimation, and behaviour classification. Detected instance segments were thereafter associated with detected skeletons based on the position of the central keypoint (withers), which was required to be located within the instance segment. Only instance segments which were associated to a single detected skeleton were retained.

After association of instance segments with detected skeletons, two metadata parameters could be determined for each segment: the animal’s orientation and behaviour. The animal’s orientation is defined as the orientation of its spine, evaluated from the camera’s perspective. Hereby, the spine is simplified to the vector from the tail implant to the withers. If the tail implant is not detected, the midpoint of the left and right hip is used as a substitute to estimate the orientation of the spine. If only one of the two hip keypoints was detected, the detected hip is used as a substitute to estimate the orientation of the spine. Using proxi-keypoints to substitute the tail implant results in a more versatile re-identification algorithm with a more general applicability. This is because the algorithm will have to cope with a higher average prediction error between the estimated and actual animal orientation. The behaviour of the animals is directly extracted from the output of the pose-estimation and behaviour classification model (Section 2.3).

**Figure 3:**
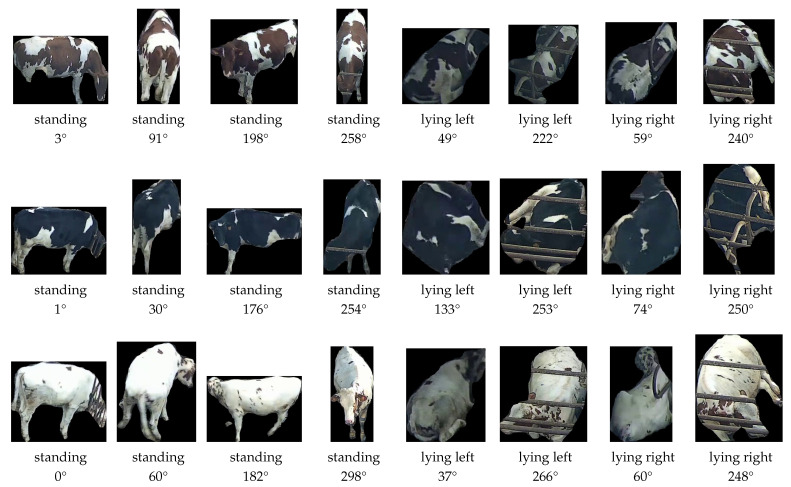
Illustration of the variation in behaviour (standing, lying left, and lying right) and orientation for three heifers (top, middle, bottom) in the dataset. The orientation is quantified based on the orientation of the spine of the animals, starting from the tail implant towards the withers.

All instance segments were manually assigned to the correct identity. During this manual step, instance segments were discarded if (1) two or more animals were present in the segment or (2) the amount of occlusion disabled manual re-identification of the animal. Furthermore, wrongly classified behaviours were corrected. The resulting dataset contains 11,438 instance segments of the 48 studied animals (https://doi.org/10.5281/zenodo.15018518). A selection of the dataset is shown for three heifers in Figure 3, illustrating the large variability in the animal’s appearance, depending on its behaviour and orientation.

### 2.5. Segment Embedding

**Figure 4:**
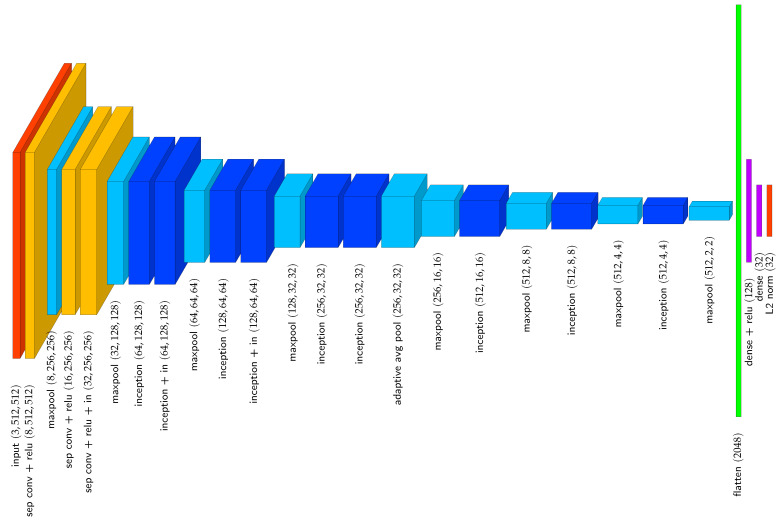
Embedding neural network used to generate 32-dimensional image embeddings. Specified tensor sizes are indicative for a 512×512 image. sep conv = separable convolution, in = instance normalisation.

**Figure 5:**
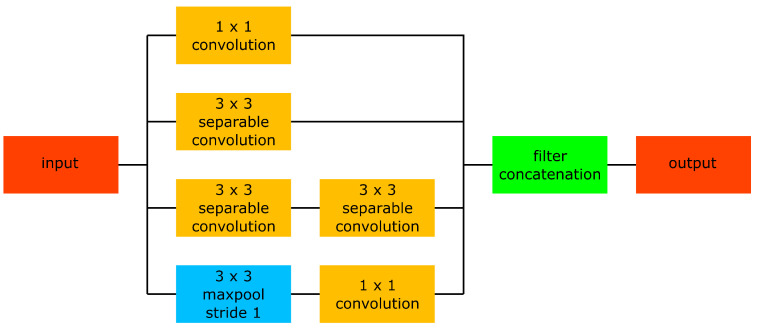
Inception module. Each operation in the inception module preserves the width and height of the input tensor. Each of the four parallel branches in the inception module generates a tensor with *c* channels, giving rise to an output tensor with 4*c* channels.

To generate embedding vectors of animal segments, we use a neural network which contains nine inception modules and is visualised in Figure 4. The architecture of the inception modules is based on Szegedy et al. [27] and is visualised in Figure 5. The output embedding vectors are 32-dimensional and L2 normalised. To evaluate the similarity between two embedding vectors, we make use of the cosine similarity Equation (2). All convolutional layers in the embedding network are separable convolutional layers [28], except the 1×1 convolutional layers within the inception modules. Furthermore, all convolutional layers use replicate padding. For regularisation purposes, three instance normalisation layers are present in the network. To generate 32-dimensional embedding vectors, independent of the width and height of the input tensor, an adaptive average pooling layer is present after the sixth inception module. The adaptive average pooling layer reshapes an input tensor of arbitrary width and height to a tensor with a width and height equal to 32.

The embedding network is trained using histogram loss [29] for 100 epochs of 1000 image batches. Batches contained 25 images and were constructed by sampling five images for five identities. Batches were constructed by first sampling five identities, a behaviour *i* and an orientation *j*, with *j* uniformly distributed over the interval $\left[0^{\circ },360^{\circ }\right]$. Thereafter, for each of the sampled identities, five instance segments at which the animal showed behaviour *i* and an orientation in the interval $\left[j-22.5^{\circ },j+22.5^{\circ }\right]$ were randomly selected. If less than five unique instances with a valid orientation were available for one or more sampled identities, the width of the valid orientation interval was gradually extended with 5° until a batch with 25 unique instance segments could be constructed. During inference, the width of the interval of valid orientations was treated as a hyperparameter and was found to be optimal when set to $90^{\circ }$. When, for benchmarking purposes, embedding networks were trained without correction for behaviour and orientation, batches were also constructed by first sampling five identities. However, thereafter, a behaviour *i* and orientation *j* were sampled five times independently for each sampled identity. Instances are thus sampled from the same overall distribution, but instances within a batch are not guaranteed to have the same behaviour and a similar orientation.

During the first 50 epochs of training, behaviours were sampled randomly. From epoch 51 on, the re-identification performance was evaluated on the validation dataset every tenth epoch, using the training dataset as a reference. Based on the average rank-1 accuracy for the different behaviours, the sampling distribution of the behaviours was altered in order to promote a more homogeneous re-identification performance across the different behaviours. Sampling probabilities for behaviour *i* were, therefore, defined proportionally to $1-{\mathrm{acc}1}_{i}$, with ${\mathrm{acc}1}_{i}$ the average rank-1 accuracy for behaviour *i*. Sampling probabilities were not allowed to take on values lower than half of the sampling probability under homogeneous sampling conditions. For our dataset, containing three behaviours, this lower bound of the sampling probabilities is 1/6.

In the default configuration of the embedding network (Figure 4), the required width *w* and height *h* of images is 512 pixels. Nevertheless, due to the adaptive average pooling layer, the embedding network can handle images of varying size. However, a constant image size of n×n is preferred to optimise re-identification performance and facilitate batch processing. Therefore, before instance segments are embedded, they are rescaled equally along both axes such that max(w,h)=n, followed by zero padding along the smallest dimension to obtain a n×n image.

During training, extensive image augmentation was performed. First, the number of identities in the training dataset was doubled by mirroring all instance segments left–right, accompanied by mirroring of the orientation and behaviours: left lying becomes right lying and vice versa. Furthermore, during the generation of training batches, colour jitter, shear transformation, small rotations ($\pm 10^{\circ }$), and scaling were deployed to augment images. Lastly, horizontal or vertical black bands were added to occlude up to 40% of the image area.

### 2.6. Re-Identification Algorithm

Before starting the re-identification of animals, a reference database is constructed containing for each animal several segments of the various possible behaviours κ∈{ll,lr,s} and with orientations θ distributed over the complete $[0^{\circ },360^{\circ }[$ interval of possible orientations (Figure 3). Each reference animal segment gives rise to a reference embedding vector, generated by using the embedding network elaborated in Section 2.5. The embedding network takes an instance segment as input and generates an L2-normalised *n*-dimensional embedding vector, with n=32.

**Figure 6:**
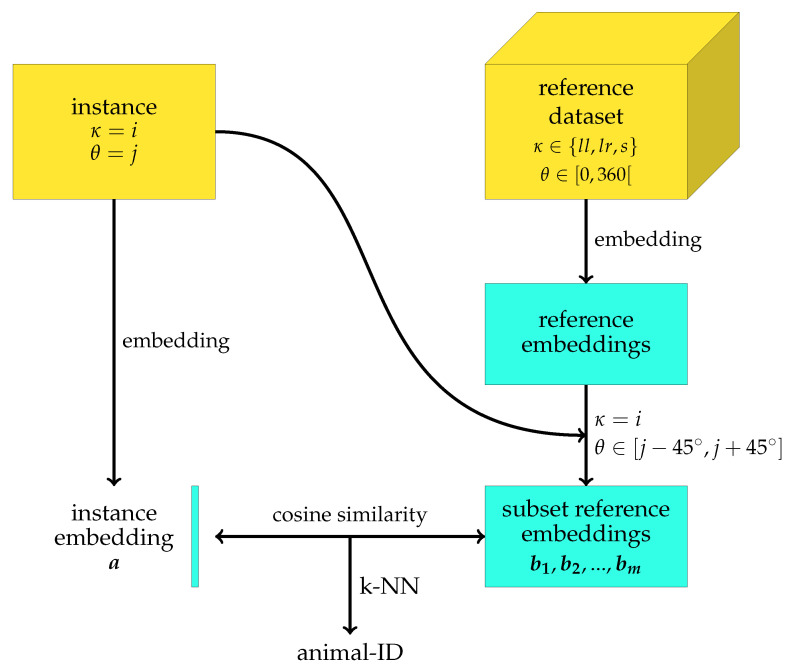
Re-identification methodology. After embedding, an instance-specific subset of the reference database is selected in which all instances show the same behaviour κ and a similar orientation θ as the inference instance. A k-NN algorithm is used to predict the identity of the inference instance, using the cosine similarity to quantify the similarity between instances.

The outline of the algorithm used during inference is shown in Figure 6 and starts by selecting a subset of the earlier constructed reference database. When an animal segment with a known behaviour κ=i and orientation θ=j has to be re-identified, an instance-specific reference dataset is created by selecting all reference embeddings associated with instances showing behaviour κ=i and having an orientation $\theta \in [j-45^{\circ },j+45^{\circ }]$. A subset of the overall reference database is thus selected for which all instances show the same behaviour and a similar orientation as the new instance that has to be re-identified.

After embedding of the new inference instance, the similarity $s_{a,b}$ of the inference embedding vector a with each of the reference embedding vectors b in the selected subset of the reference database is computed. To quantify this similarity, the cosine similarity is used, which is given by

(2) $$s_{a,b}=\frac{a\;\cdot \;b}{∥a∥∥b∥}$$

However, since all embedding vectors are L2 normalised, Equation (2) simplifies to

(3) $$s_{a,b}=a\;\cdot \;b$$

Once all similarities are obtained, a k-nearest neighbours (KNN) algorithm [30] is used to determine the most probable identity of the inference instance segment. The number of considered nearest neighbours *k* is set to $\sqrt{m}$, with *m* the cardinality of the instance-specific selected subset of the reference database.

To improve inference speed, all the reference embedding vectors $b_{1},b_{2},\ldots ,b_{m}$ in the selected subset of the reference database can be concatenated into a matrix *B*.

(4) $$B=[b_{1},b_{2},\cdots ,b_{m}]$$

This allows computing all similarities between the inference instance embedding a and the reference embeddings $b_{1},b_{2},\ldots ,b_{m}$ using the matrix product Equation (5), yielding an *m*-dimensional vector $s_{a}$ containing all relevant embedding similarities.

(5) $$s_{a}={\left[s_{a,b_{1}},s_{a,b_{2}},\ldots ,s_{a,b_{m}}\right]}^{⊤}=B^{⊤}a$$

To ameliorate the robustness of the re-identification algorithm for orientations which are only sparsely present in the reference database, we require the cardinality *m* of the selected subset to be at least $m_{min}$, with $m_{min}$ given by

(6) $$\begin{array}{cc} m_{min} & =\left\lfloor \frac{1}{4}\;\frac{90}{360}\frac{\#\mathrm{reference}\;\mathrm{embeddings}}{\#\mathrm{behaviours}}\right\rceil \\ \; & =\left\lfloor \frac{1}{16}\frac{\#\mathrm{reference}\;\mathrm{embeddings}}{\#\mathrm{behaviours}}\right\rceil \end{array}$$

$m_{min}$ thus corresponds with 25% of the expected number of reference embeddings in the selected subset if all reference embeddings would be distributed homogeneously over the different behaviours and orientations. If $m<m_{min}$, the width of the range of considered orientations $\left[j-45^{\circ },j+45^{\circ }\right]$, which has a default width of 90°, is symmetrically expanded until $m\geq m_{min}$.

### 2.7. Experimental Datasets

**Figure 7:**
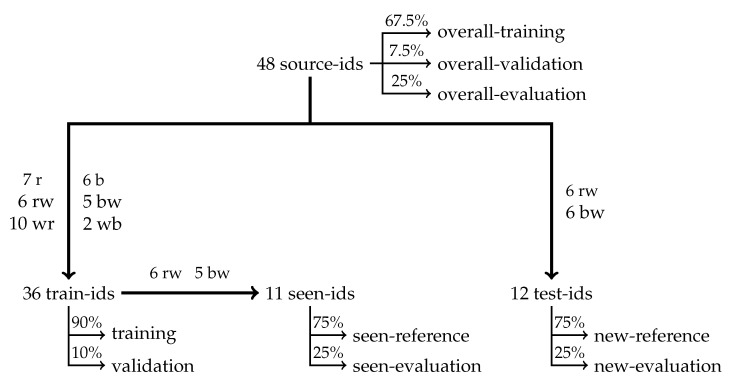
Construction of the experimental datasets.

Several datasets were constructed (Figure 7) to evaluate the re-identification performance of the developed algorithms. Starting from the 48 “source-ids” in the overall dataset, a set of test-ids was selected containing 25% of the individuals in the dataset (12). If animals would be randomly assigned to the test-ids, only a few animals of each coat-pattern group would be present in the test-ids. Consequently, a large part of the re-identification task would be reduced to classifying the coat pattern of the animals (Table 1), hence resulting in an overestimation of the actual re-identification performance. Therefore, the set of test-ids was constructed by randomly selecting six animals of each of the two most abundant coat pattern classes (rw and bw). This strategy allows for the evaluation of any re-identification algorithm with regard to its ability to distinguish animals based on specific differences in their coat patterns, rather than by quantifying the amount of white in the coat. Animal identities which were not selected as test-ids (36) were assigned to the set of train-ids. To be able to compare the re-identification performance for seen and for new animals based on datasets with a similar level of complexity, all animals of the two most abundant coat pattern classes which were not selected as test-ids (6 × rw and 5 × bw) were assigned to a set of “seen-ids”, which is thus a subset of the train-ids. Based on these three sets of identities (test-ids, seen-ids, train-ids), six datasets were created. Firstly, all instance segments available for the test-ids were randomly assigned to a new-reference database or a new-evaluation database, with 75% and 25% probability, respectively. For the animal identities in seen-ids, instance segments were randomly assigned to a seen-reference dataset or a seen-evaluation database, again with 75% and 25% probability, respectively. For the animal identities in train-ids, instance segments were randomly assigned to a training or validation dataset, with 90% and 10% probability, respectively.

Additionally, to gain more insight into which coat patterns are easier or more challenging to re-identify, an overall-evaluation dataset was constructed by selecting 25% of the instances of each individual for each behaviour. The remaining 75% of the data were used to train an overall embedding network. Similarly as described above, 10% of these images were assigned to an overall-validation dataset, while the others were assigned to the actual overall-training dataset.

### 2.8. Evaluation

After training neural networks, for each instance segment in the appropriate evaluation dataset, the procedure described in Section 2.6 is used to predict its identity. Once all instance segments in the evaluation dataset are re-identified, the re-identification performance is evaluated using the rank-*n* accuracy, with n∈{1,2,3,4,5}. The rank-*n* accuracy quantifies the fraction of all instance segments for which the actual identity is amongst the top *n*-ranked identities. The rank-1 accuracy thus quantifies the fraction of all instance segments which were correctly re-identified. In contrast, the rank-*n* accuracies with n≥2 provide more insight into why instance segments were re-identified incorrectly. The rank-*n* accuracies were determined for each behaviour separately and were thereafter averaged to obtain an overall measure of the re-identification performance. All evaluations were performed using unseen images, but if applicable, we evaluated the re-identification performance both for new (new-evaluation database) and for seen identities (seen-evaluation database), using the corresponding reference images. Moreover, embedding vectors were visualised using t-SNE plots, exploring possible patterns related to behaviour and coat pattern (Table 1). Several experiments were performed to gain more insight into the performance and added value of our proposed re-identification methodology, each of which will be elaborated below.

During the first experiment, we explored the need for a more versatile and robust re-identification algorithm. Therefore, embedding-based re-identification was performed without correcting for behaviour and orientation. During this experiment, our embedding network was trained on our dataset and on the OpenCows dataset of Andrew et al. [14], which contains only top-view images of standing cows. In the subsequent experiment, we quantified the added value of correction for behaviour and orientation by training and evaluating our embedding network with and without correction for behaviour and orientation.

In the third experiment, our embedding network was trained on the overall-training dataset, which contains all 48 “source-identities”. During this experiment, the network was evaluated with regard to its within coat-pattern group re-identification performance, as well as with regard to its overall re-identification performance for seen identities.

Benchmarking happened during the fourth experiment, in which our network architecture was compared to several well-known backbones: Alexnet [31], InceptionV3 [27], and ResNet-50 [32]. Simultaneously, the (potentially) added value of pre-training on ImageNet [33] was explored.

Lastly, a sensitivity analysis was performed regarding the input image resolution, the network capacity, and the training dataset size. To evaluate the influence of the input image resolution, additional embedding networks were trained and evaluated on images with 64, 128, and 256 pixels width and height instead of the default 512 pixels. To evaluate the influence of network capacity on the re-identification performance, networks were trained for which the tensor generated by the last inception layer has 64, 128, 256, 512, and 1024 channels. The depth of the output tensors generated by the first eight inception modules was hereby changed proportionally to the change in the depth of the tensor generated by the last inception layer. Finally, embedding networks were trained with data of 12, 24, and 36 identities to explore the impact of the training dataset size.

## 3. Results and Discussion

Table 2 shows the rank-1 and rank-2 re-identification accuracy for re-identification without correction for the behaviour and orientation of the animals. Moreover, the rank-1 and rank-2 accuracies for seen and new individuals are compared between our dataset and the OpenCows dataset of Andrew et al. [14]. Our embedding-based re-identification method applied to the OpenCows dataset delivers an excellent rank-1 accuracy of 0.985 for seen identities. For new identities, the rank-1 accuracy drops slightly to 0.959. However, the rank-2 accuracy is still 0.990, which indicates that most wrongly identified images result from confusion between two animals that are probably quite similar and not due to a complete failure of re-identification. The rank-1 re-identification results for seen identities in our dataset are similar to the rank-1 accuracy for new identities in the OpenCows dataset. However, for new identities in our dataset, the average rank-1 accuracy reduces to 0.822. Note the impact of the behaviour: for standing animals, the rank-1 accuracy is severely reduced to 0.777, while the rank-1 accuracy for animals lying right is still 0.890. Moreover, it takes until the rank-4 accuracy to reach a value above 0.99, indicating that a significant part of the wrongly re-identified animals is not a result of confusion between two similar individuals, but rather a complete failure to re-identify animal segments.

The large drop in rank-1 accuracy on our dataset for new identities suggests that the embedding network learns the patch pattern of each identity in the training dataset by heart. This allows the embedding network to generate similar embedding vectors for two instance segments of the same animal, even if the two images have little in common. As a result, the rank-1 accuracy for seen identities is still good. However, this strategy does not generalise, leading to a severe drop in performance when re-identification has to be performed for new identities.

To improve the re-identification performance for unseen identities, we proposed to use metadata about the behaviour and orientation of the animal to construct a more homogeneous reference dataset for each animal segment which has to be re-identified. The results of applying this correction for orientation and behaviour are shown in Table 3. When comparing the rank-1 accuracy with and without correction for behaviour and orientation, one can observe a significant improvement in the rank-1 accuracy with 0.072. This improvement corresponds with a 40% decrease in the number of animal segments which is wrongly re-identified. Moreover, the improvement in the rank-1 accuracy is present for all behaviours. For lying left and standing, the two behaviours with a rank-1 accuracy below 0.80, the rank-1 accuracy improves with about 0.08. For lying right, on the other hand, the improvement is with 0.052 slightly lower. Nevertheless, as the rank-1 accuracy for lying right without correction for behaviour and orientation was already 0.890, a lower improvement could be expected. If the performance improvement is, however, expressed in terms of the relative decrease in the number of wrongly re-identified instances, the performance improvement for lying left, lying right, and standing are 42%, 47%, and 36%, respectively. From this perspective, the performance improvement for lying right is even the largest of the three considered behaviours.

The differences in rank-1 accuracy between the different behaviours should, however, be interpreted carefully. From Table 3, one could, for example, conclude that re-identification of animals lying right is significantly easier compared to the re-identification of animals lying left. However, this is not true. If one would mirror all instance segments left/right, this would result in the opposite conclusion, while both scenarios could be valid in the real world. The main cause for the differences in rank-1 accuracy between the different behaviours originates in the modest size of our test dataset, even though the number of identities in our test dataset is similar to those of test datasets used in previous research on animal re-identification [14,16,18]. Knowing that for animals lying left, mostly the right flank is visible, while for animals lying right, mostly the left flank is visible, the differences in rank-1 accuracy between different behaviours rather indicate that, by chance, there are more groups of several animals in our dataset which have a similar right flank than groups of animals having a similar left flank.

Table 4 shows the rank-1 accuracies realised by the embedding network trained on all 48 identities in our dataset. This model was evaluated by determining its rank-1 accuracy with respect to the within coat-pattern group (Table 1) re-identification for seen identities. Additionally, the overall rank-1 accuracy for re-identification of seen identities was obtained by using all 48 identities simultaneously. With regard to the within coat-pattern group rank-1 re-identification accuracy, the average rank-1 accuracy is approximately the same for r, b, rw, and rb (all 0.95–0.96) and similar to the results reported for seen identities in Table 3. The rank-1 accuracies for the separate behaviours show larger differences, but one should consider that these rank-1 accuracies have larger estimation variances compared to the average rank-1 accuracy. For wb, all rank-1 accuracies are 1, but since this group contains only two individuals, which apparently are fairly easy to distinguish from each other, no conclusions may be drawn from this result. The wr coat-pattern group, on the other hand, reports the lowest rank-1 accuracy of all coat-pattern groups, which is about 0.08 lower than the average rank-1 accuracy of the other coat-pattern groups. This is probably because the animals in the wr class often had only a few small red patches. If several of these red patches are occluded by other animals or by the pose of the animal itself, the segmentation algorithm results in segments that barely contain any coloured patches. During annotation, we found these segments the most difficult to assign to a correct identity, since sometimes only a subtle difference in the shape of a single visible red patch delivered the necessary information to assign a segment to its correct identity. From the results in Table 4, it can be observed that the embedding model is also struggling to some extent with this complexity. It could be argued why the same reasoning does not hold for the r and b coat-pattern groups, since all four categories r, b, wr, and wb, have in common that one of the two coat colours is present for less than 20% of the coat area. However, the few white patches of the r and b animals were nearly always located around the spine of the animals and had mostly an elongated shape. This is in contrast to the wr animals, where the red patches were mainly located at the flanks and generally had a more rounded shape. Since (1) patches located around the spine are occluded less frequently compared to patches located further from the spine and (2) more elongated patches are easier to distinguish from each other compared to rounded patches, these differences in the location and shape of coat patches are possible reasons for the observed differences in the rank-1 accuracy between the wr coat pattern group on the one hand, and the r and b coat-pattern groups on the other hand.

The rank-1 accuracies for re-identification of all 48 seen identities simultaneously are shown at the bottom of Table 4. The average rank-1 re-identification accuracy of 0.912 is significantly lower compared to the average rank-1 re-identification accuracy of 0.971 which is reported in Table 3 for seen animals when correcting for behaviour and orientation, as well as all but one of the other reported average rank-1 accuracies in Table 4. This observation is expected as an increased number of possible identities goes hand in hand with a higher probability of the model being confused between two or more identities for a given animal segment. While the reported overall rank-1 re-identification accuracy is significantly lower than the value of 0.971 reported in Table 3, the differences in the average rank-2 accuracy are much more modest: 0.972 versus 0.982 for re-identification of seen identities with 48 versus 11 possible identities. This indicates that the embedding network is more often confused between two similar individuals when the number of possible identities increases, but that the number of totally wrong re-identifications stays about the same. Compared to the small colour pattern groups with maximum 12 identities, the set of 48 possible identities is more in line with commercial group sizes of lactating dairy cows. Therefore, the obtained rank-1 re-identification accuracy for the overall group with 48 seen identities gives a better indication of the rank-1 re-identification accuracy that can be expected in practical applications. When herd sizes become even larger, up to several hundreds of animals, as is common in some parts of the world, one can expect two counteracting effects influencing the rank-1 re-identification accuracy. On the one hand, with an increasing number of animals, there is an increase in the probability of two animals having a similar coat pattern, lowering the rank-1 re-identification accuracy. On the other hand, if all animals of the herd would (initially) be used to train the re-identification algorithm, larger herd sizes will result in higher quality discriminative features being extracted by the embedding network, as will be shown later in this paper (Figure 13). The latter effect on the rank-1 re-identification accuracy will partially compensate the former, but to quantify this compensation, further research is required.

When multiple animals are present on a single camera frame, maximising the joint probability, computed by multiplication of the identity probabilities for all detected instances, offers an opportunity to leverage the rank-1 re-identification accuracy. This joint probability should be maximised under the condition that each detected instance has to be assigned a unique identity, since it is evident that an animal cannot be present twice in a single frame. This approach will reduce the probability of incorrect identification when two regularly confused animals are present simultaneously in the camera’s field of view. Hence, the average rank-1 re-identification accuracy is expected to improve. This principle of joint probability maximisation can be extended to scenarios in which animals have to be re-identified subsequently, for example, in the milking parlour. Whenever a new animal enters the milking parlour, it is possible to maximise the joint identity probability of the current and all previous animals, since each animal is expected to pass only once. Using this joint re-identification approach instead of re-identifying every animal independently, will reduce the number of incorrect re-identifications.

In most herds, new animals enter the herd on a weekly or even on a daily basis. While our embedding-based methodology is readily extendable to new identities without retraining of the embedding network, it may be evident from Table 3 that the re-identification performance for seen identities will always be higher than for new identities. However, retraining/finetuning the embedding network for each animal entering the herd would strongly increase the energy consumption, and thus cost, of a computer vision-based re-identification algorithm. Moreover, retraining/finetuning the embedding network requires more costly hardware than is required for inference. Therefore, we suggest to apply periodically (remote) finetuning, for example, monthly, to mitigate the cost of energy consumption, while simultaneously consolidating to a large extent the improved re-identification performance for seen identities.

**Figure 8:**
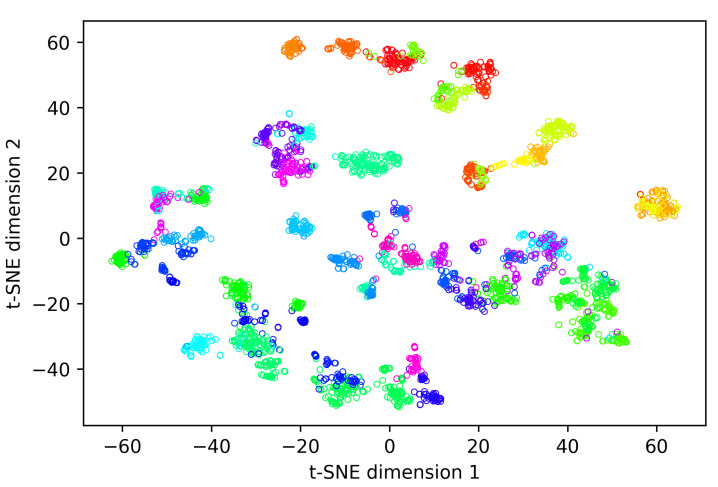
T-SNE visualisation of the embedding vectors generated for the overall test dataset. Different symbol colours indicate different identities.

Figure 8 shows the first two components of a t-SNE analysis of the embeddings generated while evaluating our re-identification methodology on the overall-evaluation dataset containing all 48 different identities present in the dataset. During this experiment, an average rank-1 re-identification accuracy of 0.912 was realised (Table 4). In Figure 8, one can observe that the embedding vectors of most individuals form clear clusters. This is remarkable since our methodology does not require generating similar embeddings for all of the animal’s possible behaviours and orientations to achieve a good rank-1 re-identification accuracy. On the other hand, coat pattern properties such as patch size and patch edging (smooth vs. irregular) are often quite similar over the whole body. Therefore, it is not surprising the embedding network generates similar embeddings for different behaviours and orientations of an animal.

**Figure 9:**
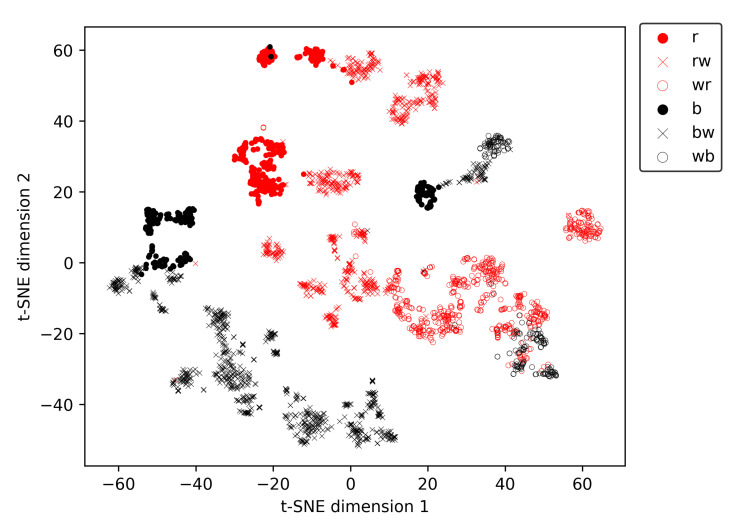
T-SNE visualisation of the embedding vectors generated for the overall test dataset, stratified by coat pattern. Different symbol colours indicate a different animal colour (black vs. red), different symbols indicate a different amount of white in the coat. r = red, rw = red and white, wr = white and red, b = black, bw = black and white, wb = white and black.

To explore the presence of metaclusters related to the coat pattern in the t-SNE vectors, we created Figure 9, in which the marker type and colour are depending on the animal’s coat pattern (Table 1). Ignoring the amount of white in the coats for a moment, three clear metaclusters are present in Figure 9, each predominantly containing animals of a single hair colour. The first metacluster is located at the bottom-left of Figure 9 and contains only black-haired animals. The second large metacluster is banana-shaped and located more centrally in the figure. This metacluster contains predominantly red-haired animals, together with instances of a single animal with a wb coat pattern. The third and smallest metacluster is positioned in the middle of the previously described banana-shaped metacluster and contains only black-haired animals. The single black-haired animal in the second, predominantly red-haired, metacluster is an almost entirely white animal with only a few small black patches, of which none have a diameter larger than 20 cm. Therefore, it is no surprise this animal ends up in the predominantly red-haired cluster. After all, there are several red-haired individuals which also have only a few small red patches in their coat, while the other wb animal in the dataset has much larger, more elongated black patches, with dimension well over 50 cm. In each metacluster, the amount of white in the coat increases along the main axis of the metacluster. For example, for the predominantly red-haired metacluster, all predominantly red-coloured animals are located at the top-left of the metacluster. More towards the right/bottom of the metacluster, the red and white (rw) coloured animals are located, while all the white and red (wr) animals are situated at the bottom-right part of the metacluster.

**Figure 10:**
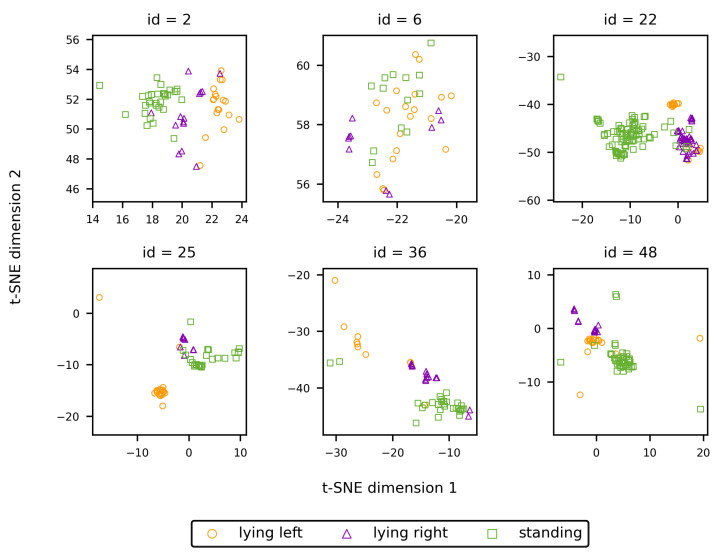
T-SNE vectors for some randomly selected identities, stratified according to the shown behaviour.

Figure 10 allows to study the embeddings of individual animals with regard to the presence of behaviour-related clusters. For most identities, some degree of clustering according to the shown behaviour is present. This apparent clustering originates from the training procedure of the embedding network, during which batches are constructed conditional on the animal’s behaviour. As a consequence, the incentive of the embedding network to generate similar embeddings for images of a single identity is also conditional on the animal’s behaviour. However, since the properties of the coat pattern are quite similar over the whole body, as mentioned earlier, similar embeddings are generated for the different behaviours. Hence, the mutual position of the cluster centres is only defined by randomness and is of no influence on the re-identification performance.

In Table 5, the rank-1 re-identification results are shown for new identities using various backbone architectures. Furthermore, the usage of ImageNet pre-trained weights was compared with training from scratch. From this table, it is clear that our model architecture has by far the lowest number of parameters. While Inception V3 and ResNet-50 both have slightly more than 25,000 k parameters and AlexNet even has 57,134 k parameters, our network architecture has only 1160 k parameters, which is only 4.5% of the number of parameters in Inception V3. Nevertheless, nearly all models achieve similar rank-1 accuracies, varying between 0.869 and 0.894. The only exception is the Inception V3 architecture without using ImageNet pre-trained initial weights, using this backbone results in an average rank-1 accuracy of only 0.537. This rank-1 accuracy is better than the trivial rank-1 accuracy of 1/12 that would be expected in the case of random guessing, but it does not result in a re-identification model useful for practical applications. When weight initialisation is performed using ImageNet pre-trained weights, the Inception V3 backbone delivers similar results as the other backbones, indicating the network capacity is not at all an issue in restricting the realised rank-1 re-identification accuracy. Moreover, the number of inception modules in Inception V3 is quite similar to the number of inception modules in our network: 11 vs. 9. However, the design of our inception modules is slightly different from those of Inception V3 and we only use separable convolutional layers instead of the ordinary conventional convolutional layers in Inception V3. The results in Table 5 suggest that these differences allow our embedding network to be easily trained from scratch, while this is not the case for the Inception V3 backbone. For the benchmark backbones that deliver decent results when trained from scratch (AlexNet and Resnet-50), the rank-1 accuracy does not improve significantly when using Imagenet pre-trained weights instead of randomly initialised weights. The average rank-1 accuracy for AlexNet improved slightly, while the average rank-1 accuracy for ResNet-50 slightly decreased. Moreover, the differences in the rank-1 accuracies for the individual behaviours are not coherent: some do improve, some stay about the same, and some even decline. This observation is in contrast with the results shown in Table 3, where the rank-1 accuracies for new identities coherently increase for all behaviours. Therefore, it can be concluded that if a neural network converges without using Imagenet pre-trained initial weights, there is little performance improvement to be expected by using Imagenet pre-trained initial weights.

**Figure 11:**
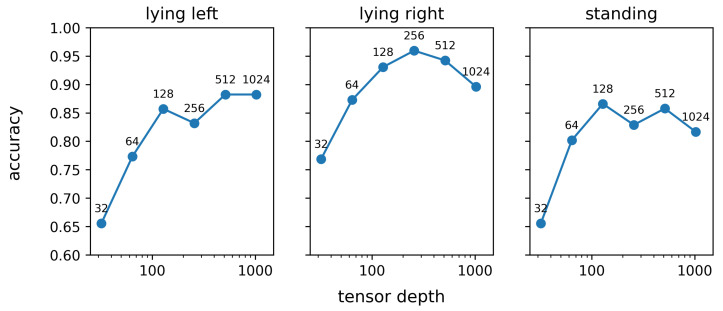
Effect of the tensor depth after the last inception module on the rank-1 re-identification accuracy.

Figure 11 shows the result of the sensitivity analysis that was performed to evaluate the impact of the network capacity on the rank-1 re-identification performance. In Figure 11, the network capacity is quantified by the depth of the tensor obtained after application of the last inception module, as elaborated in Section 2.8. For all three studied behaviours, the rank-1 re-identification accuracy monotonously increases when the tensor depth after the last inception module increases from 32 to 128. However, a further increase in network capacity does not result in an improved re-identification performance. The average rank-1 re-identification for tensor depths of 128, 256, 512, and 1024 is relatively constant and varies between 0.87 and 0.89. The trends in the rank-1 re-identification performance in Figure 11 for tensor depths over 128 are, therefore, mainly due to coincidence and are not coherent over the different behaviours. This observation aligns with the results in Table 5, in which network architectures with more parameters, and thus a higher network capacity, do not result in an improved re-identification performance. On the other hand, the results visualised in Figure 11 clearly show that below a certain threshold, lowering the network capacity inevitably results in a decrease in the re-identification performance. Based on our results, the depth of the tensor resulting from the last inception layer can thus be reduced up to 128 without any significant loss of performance. However, it is important to consider that making the network more lightweight in this way will reduce the computer memory requirements and energy consumption, but will not improve the inference speed.

**Figure 12:**
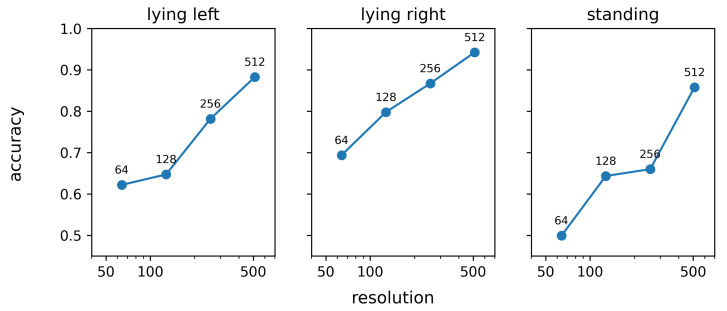
Effect of the image resolution, expressed as the number of pixels across the width and height, on the rank-1 re-identification accuracy.

Figure 12 shows the influence of the image resolution on the rank-1 re-identification performance. This figure clearly shows that a higher image resolution results in an improved re-identification performance. This observation can be explained based on the median width and height of the images in the test database, which is 297 and 263 pixels, respectively. When, for example, a 256 × 256 input image is used instead of the default 512 × 512 input image, more than half of the images have to be scaled down. The loss of resolution following from this downscaling results in a loss of apparently useful information and hence also in a loss of rank-1 re-identification performance. Given the 95% percentile of the image width and height, which is 469 and 398 pixels, respectively, it can be expected that the performance will have reached its maximum for the 512 × 512 images. Therefore, using larger input images will not lead to an improved re-identification performance.

**Figure 13:**
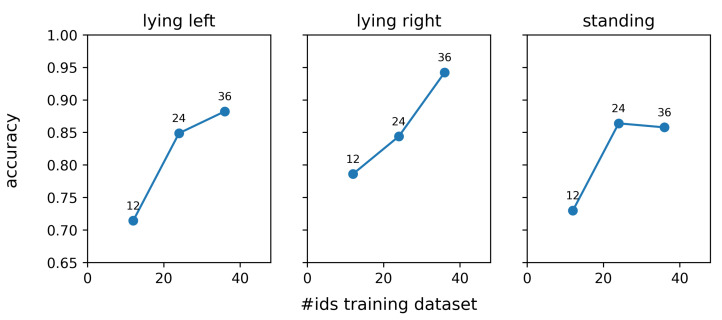
Effect of the number of identities in the training dataset on the rank-1 re-identification accuracy.

Figure 13 shows the influence of the training dataset size on the re-identification performance. For each data point in this figure, the same test dataset was used, containing 12 individuals, of which six are red and white (rw) and six are black and white (bw). It is clear from the results that a larger training dataset results in an improved re-identification performance. Adding more individuals to our dataset could thus improve the re-identification performance further. However, one has to consider that the marginal increment in the overall rank-1 re-identification performance will decrease with increasing dataset size. Previous exploratory research revealed that the amount of overfitting of the embedding network is relatively limited, and therefore, other factors are responsible for the observed performance increment. One possible reason is that the addition of new individuals to the dataset results in the presence of more similar individuals and hence requires the network to construct more efficient features.

It should be noted that the size of the training dataset reported in Figure 13 cannot be compared directly to the datasets used in previous publications on animal re-identification [14,18], where re-identification was performed for a single pose only with standardised camera perspective, e.g., a top-down view of standing animals. In those previous studies, the number of actual individuals equals the number of identities “perceived” by the neural network during training. This apparently trivial assumption, however, does not hold for our research. In theory, by distinguishing three different behaviours and sampling batches from randomly selected orientation intervals of 45°, a single actual individual gives rise to 3×8=24 fictive individuals perceived by the network. Moreover, since the set of mirrored instances of each individual was included in the dataset as a new unique individual, the number of fictive individuals per actual individual doubles once more and becomes 48. However, in practice, the orientations of animals lying right or lying left will show a strongly bimodal distribution and, therefore, the usage of orientation intervals of 45° gives rise to eight fictive individuals for standing, two fictive individuals for lying left, and two fictive individuals for lying right, instead of eight fictive individuals for each behaviour. This results in a total of only 2×(8+2+2)=24 fictive individuals per actual individual. Since our complete training dataset contained 36 actual individuals, this corresponds to a total of 864 fictive individuals. Nevertheless, during evaluation, the number of individuals the algorithm has to distinguish is always 12, the number of actual individuals in the test dataset. Hence, the results presented in this paper can be considered as being a result of training an embedding network using a dataset of 864 (fictive) individuals and subsequently evaluating the obtained network on a test dataset containing 12 individuals.

Despite the fact that our methodology removes several hurdles for the application of computer vision-based re-identification in commercial environments, some challenges still remain. First of all, we showed that the fraction of the coat which is white is an important discriminative feature to assign the correct identity to an animal. The simultaneous presence of white and coloured patches, with a high inter-individual variation, is a typical characteristic of pied cattle breeds, such as Holstein and Fleckvieh. However, several other cattle breeds, such as Jersey, Brown Swiss, and Angus, have a homogeneous coat. It can be expected that computer vision-based re-identification will be considerably more difficult for these breeds. Secondly, our algorithm was only trained using daylight images. However, daylight images are more informative compared to night vision images, since the latter lack colour information and contain often less contrast. Therefore, night vision images are expected to result in a decrease in the re-identification performance.

## 4. Conclusions

State-of-the-art methods for animal re-identification require a standardised animal pose. However, this requirement currently limits the practical applicability of computer vision-based re-identification. To address this, we propose an alternative approach that combines image embeddings with additional metadata. More specifically, we employ a second neural network to determine the animals’ behaviour and orientation. By using this additional information, we are able to create a tailored reference dataset for each inference instance. We accomplish this by retaining only instances from the overall reference dataset that show the same behaviour and a similar orientation. Without considering any additional information from the metadata parameters, we achieved a rank-1 accuracy of only 0.822 for our dataset. However, when the behaviour and orientation of the animals were taken into account, the rank-1 accuracy increased to 0.894, corresponding to a 40% reduction in incorrectly identified animals.

## Figures and Tables

**Table 1:** Frequency of coat patterns in the dataset.

Coat Pattern	Abbrv.	#Ids
red	r	7
black	b	6
red and white	rw	12
black and white	bw	11
white and red	wr	10
white and black	wb	2
overall		48

**Table 2:** Rank-1 and rank-2 re-identification accuracy without correction for the behaviour and orientation of the animals. The OpenCows dataset [14] contains only top-view images of standing animals. Our dataset contains images of animals lying left, lying right, and standing, observed with varying animal orientations. Below every reported value, the corresponding 95% confidence interval is given.

Rank	Dataset	Ids	#Ids	Lying Left	Lying Right	Standing	Avg.
1	OpenCows	seen	34	-	-	0.985 [0.968, 0.994]	0.985 [0.968, 0.994]
		new	12	-	-	0.959 [0.899, 0.989]	0.959 [0.899, 0.989]
	Ours	seen	11	0.987 [0.955, 0.999]	0.952 [0.914, 0.977]	0.948 [0.921, 0.968]	0.963 [0.955, 0.971]
		new	12	0.798 [0.715, 0.866]	0.890 [0.834, 0.933]	0.777 [0.738, 0.814]	0.822 [0.805, 0.839]
2	OpenCows	seen	34	-	-	0.995 [0.982, 0.999]	0.995 [0.982, 0.999]
		new	12	-	-	0.990 [0.945, 0.999]	0.990 [0.945, 0.999]
	Ours	seen	11	0.987 [0.955, 0.999]	0.990 [0.966, 0.999]	0.987 [0.970, 0.996]	0.988 [0.983, 0.993]
		new	12	0.924 [0.861, 0.965]	0.965 [0.926, 0.987]	0.934 [0.908, 0.954]	0.941 [0.930, 0.952]

**Table 3:** Effect of correction for behaviour (κ) and orientation (θ) on the rank-1 and rank-2 re-identification accuracy for seen and new animals. Below every reported value, the corresponding 95% confidence interval is given. ∼(κ,θ) = correction for behaviour and orientation.

Rank	Ids	#Ids	∼(κ,θ)	Lying Left	Lying Right	Standing	Avg.
1	seen	11	no	0.987 [0.955, 0.999]	0.952 [0.914, 0.977]	0.948 [0.921, 0.968]	0.963 [0.955, 0.971]
			yes	0.987 [0.955, 0.999]	0.957 [0.920, 0.980]	0.969 [0.946, 0.984]	0.971 [0.964, 0.978]
	new	12	no	0.798 [0.715, 0.866]	0.890 [0.834, 0.933]	0.777 [0.742, 0.817]	0.822 [0.805, 0.839]
			yes	0.882 [0.811, 0.934]	0.942 [0.896, 0.972]	0.858 [0.823, 0.888]	0.894 [0.880, 0.908]
2	seen	11	no	0.987 [0.955, 0.999]	0.990 [0.966, 0.999]	0.987 [0.970, 0.996]	0.988 [0.983, 0.993]
			yes	0.994 [0.966, 0.999]	0.967 [0.933, 0.987]	0.984 [0.966, 0.994]	0.982 [0.977, 0.987]
	new	12	no	0.924 [0.861, 0.965]	0.965 [0.926, 0.987]	0.934 [0.908, 0.954]	0.941 [0.930, 0.952]
			yes	0.958 [0.905, 0.986]	0.994 [0.968, 0.999]	0.957 [0.935, 0.973]	0.970 [0.963, 0.977]

**Table 4:** Rank-1 re-identification accuracy for within colour-pattern group re-identification of seen individuals. Below every reported value, the corresponding 95% confidence interval is given.

Colour Pattern	#Ids	Lying Left	Lying Right	Standing	Avg.
red	7	0.986 [0.926, 0.999]	1.000 [0.926, 1.000]	0.894 [0.839, 0.935]	0.960 [0.952, 0.968]
black	6	0.926 [0.821, 0.980]	1.000 [0.940, 1.000]	0.923 [0.863, 0.963]	0.950 [0.937, 0.963]
red and white	12	0.927 [0.876, 0.962]	0.985 [0.946, 0.998]	0.958 [0.933, 0.976]	0.957 [0.948, 0.966]
black and white	11	0.975 [0.929, 0.995]	0.976 [0.948, 0.991]	0.905 [0.875, 0.931]	0.952 [0.944, 0.960]
white and red	10	0.882 [0.787, 0.944]	0.890 [0.830, 0.935]	0.836 [0.794, 0.872]	0.869 [0.851, 0.887]
white and black	2	1.000 [0.916, 1.000]	1.000 [0.946, 1.000]	1.000 [0.953, 1.000]	1.000 [0.979, 1.000]
overall	48	0.934 [0.909, 0.954]	0.920 [0.897, 0.939]	0.883 [0.866, 0.899]	0.912 [0.906, 0.918]

**Table 5:** Rank-1 re-identification accuracy using different backbone architectures. Initial weights were based on ImageNet pre-training or were randomly generated.

Initial Weights	Architecture	# Params.	Lying Left	Lying Right	Standing	Avg.
ImageNet	AlexNet	57,134 k	0.857	0.942	0.874	0.891
	Inception V3	25,178 k	0.832	0.942	0.909	0.894
	ResNet-50	25,574 k	0.891	0.873	0.849	0.871
-	AlexNet	57.134 k	0.815	0.919	0.872	0.869
	Inception v3	25,178 k	0.563	0.566	0.482	0.537
	ResNet-50	25,574 k	0.840	0.896	0.901	0.879
	Ours	1160 k	0.882	0.942	0.858	0.894

## Data Availability

The data that support the findings of this study are openly available in zenodo at https://doi.org/10.5281/zenodo.14975828, https://doi.org/10.5281/zenodo.15016853 and https://doi.org/10.5281/zenodo.15018518.

## References

[1] Bowling M.B., Pendell D.L., Morris D.L., Yoon Y., Katoh K., Belk K.E., Smith G.C. (2008). Identification and traceability of cattle in selected countries outside of North America. Prof. Anim. Sci..

[2] Lopes M.A., Junqueira L.V., Bruhn F.R.P., Demeu A.A., das Dores Silva M. (2017). Technical efficiency and economic viability of different cattle identification methods allowed by the Brazilian traceability system. Semin. Ciências Agrárias Londrina.

[3] Bagnato S., Pedruzzi L., Goracci J., Palagi E. (2023). The interconnection of hierarchy, affiliative behaviours, and social play shapes social dynamics in Maremmana beef cattle. Appl. Anim. Behav. Sci..

[4] Briefer E.F., Sypherd C.C.R., Linhart P., Leliveld L.M.C., Padilla de La Torre M., Read E.R., Guérin C., Deiss V., Monestier C., Rasmussen J.H. (2022). Classification of pig calls produced from birth to slaughter according to their emotional valence and context of production. Sci. Rep..

[5] Tallet C., Linhart P., Policht R., Hammerschmidt K., Šimeček P., Kratinova P., Špinka M. (2013). Encoding of situations in the vocal repertoire of piglets (Sus scrofa): A comparison of discrete and graded classifications. PLoS ONE.

[6] Bouissou M.F. (1980). Social relationships in domestic cattle under modern management techniques. Ital. J. Zool..

[7] Zheng L., Shen L., Tian L., Wang S., Wang J., Tian Q. Scalable Person Re-Identification: A Benchmark. Proceedings of the IEEE International Conference on Computer Vision.

[8] Ristani E., Solera F., Zou R., Cucchiara R., Tomasi C. Performance Measures and a Data Set For multi-Target, Multi-Camera Tracking. Proceedings of the European Conference on Computer Vision.

[9] Wei L., Zhang S., Gao W., Tian Q. Person Transfer GAN to Bridge Domain Gap for Person Re-Identification. Proceedings of the IEEE Conference on Computer Vision and Pattern Recognition (CVPR 2018).

[10] Chen G., Gu T., Lu J., Bao J.A., Zhou J. (2021). Person re-identification via attention pyramid. IEEE Trans. Image Process..

[11] Wang G., Yuan Y., Chen X., Li J., Zhou X. Learning Discriminative Features with Multiple Granularities for Person Re-Identification. Proceedings of the 26th ACM International Conference on Multimedia.

[12] Zhang F., Ma H., Zhu J., Hamdulla A., Zhu B. (2024). FRCE: Transformer-based feature reconstruction and cross-enhancement for occluded person re-identification. Expert Syst. Appl..

[13] Fu D., Chen D., Bao J., Yang H., Yuan L., Zhang L., Li H., Chen D. Unsupervised Pre-Training for Person Re-Identification. Proceedings of the IEEE/CVF Conference on Computer Vision and Pattern Recognition.

[14] Andrew W., Gao J., Mullan S., Campbell N., Dowsey A.W., Burghardt T. (2021). Visual identification of individual Holstein-Friesian cattle via deep metric learning. Comput. Electron. Agric..

[15] Deb D., Wiper S., Gong S., Shi Y., Tymoszek C., Fletcher A., Jain A.K. Face Recognition: Primates in the Wild. Proceedings of the 2018 IEEE 9th International Conference on Biometrics Theory, Applications and Systems (BTAS).

[16] Bergamini L., Porrello A., Dondona A.C., Del Negro E., Mattioli M., D’alterio N., Calderara S. Multi-Views Embedding for Cattle Re-Identification. Proceedings of the 2018 14th International Conference on Signal-Image Technology & Internet-Based Systems (SITIS 2018).

[17] Dac H.H., Gonzalez Viejo C., Lipovetzky N., Tongson E., Dunshea F.R., Fuentes S. (2022). Livestock identification using deep learning for traceability. Sensors.

[18] Zin T.T., Phyo C.N., Tin P., Hama H., Kobayashi I. Image Technology Based Cow Identification System Using Deep Learning. Proceedings of the International MultiConference of Engineers and Computer Scientists.

[19] Xiao J., Liu G., Wang K., Si Y. (2022). Cow identification in free-stall barns based on an improved Mask R-CNN and an SVM. Comput. Electron. Agric..

[20] Andrew W., Greatwood C., Burghardt T. Visual Localisation and Individual Identification of Holstein Friesian Cattle via Deep Learning. Proceedings of the IEEE International Conference on Computer Vision Workshops.

[21] Schroff F., Kalenichenko D., Philbin J. Facenet: A Unified Embedding for Face Recognition and Clustering. Proceedings of the IEEE Conference on Computer Vision and Pattern Recognition.

[22] He K., Gkioxari G., Dollár P., Girshick R. Mask r-cnn. Proceedings of the IEEE International Conference on Computer Vision.

[23] Perneel M., Adriaens I., Aernouts B., Verwaeren J. (2025). Consistent multi-animal pose estimation in cattle usingdynamic Kalman filter based tracking. arXiv.

[24] Schneider D., Lindner K., Vogelbacher M., Bellafkir H., Farwig N., Freisleben B. (2024). Recognition of European mammals and birds in camera trap images using deep neural networks. IET Comput. Vis..

[25] Elhamod M., Diamond K.M., Maga A.M., Bakis Y., Bart H.L., Mabee P., Dahdul W., Leipzig J., Greenberg J., Avants B. (2022). Hierarchy-guided neural network for species classification. Methods Ecol. Evol..

[26] Dyrmann M., Karstoft H., Midtiby H.S. (2016). Plant species classification using deep convolutional neural network. Biosyst. Eng..

[27] Szegedy C., Vanhoucke V., Ioffe S., Shlens J., Wojna Z. Rethinking the Inception Architecture for Computer Vision. Proceedings of the IEEE Conference on Computer Vision and Pattern Recognition.

[28] Chollet F. Xception: Deep Learning with Depthwise Separable Convolutions. Proceedings of the IEEE Conference on Computer Vision and Pattern Recognition.

[29] Ustinova E., Lempitsky V. Learning Deep Embeddings with Histogram Loss. Proceedings of the 30th Conference on Neural Information Processing Systems (NIPS).

[30] Hastie T., Tibshirani R., Friedman J. (2008). The Elements of Statistical Learning.

[31] Krizhevsky A., Sutskever I., Hinton G.E. (2012). Imagenet classification with deep convolutional neural networks. Advances in Neural Information Processing Systems 25 (NIPS 2012).

[32] He K., Zhang X., Ren S., Sun J. Deep Residual Learning for Image Recognition. Proceedings of the IEEE Conference on Computer Vision and Pattern Recognition.

[33] Deng J., Dong W., Socher R., Li L.J., Li K., Fei-Fei L. Imagenet: A large-Scale Hierarchical Image Database. Proceedings of the 2009 IEEE Conference on Computer Vision and Pattern Recognition.

